# Growth mindset and school burnout symptoms in young adolescents: the role of vagal activity as potential mediator

**DOI:** 10.3389/fpsyg.2023.1176477

**Published:** 2023-07-13

**Authors:** Smiddy Nieuwenhuis, Denise J. van der Mee, Tieme W. P. Janssen, Leonie L. L. Verstraete, Martijn Meeter, Nienke M. van Atteveldt

**Affiliations:** ^1^Section Clinical, Neuro and Developmental Psychology, Faculty of Behavioral and Movement Science, Vrije Universiteit Amsterdam, Amsterdam, Netherlands; ^2^Institute for Brain and Behavior Amsterdam, Vrije Universiteit Amsterdam, Amsterdam, Netherlands; ^3^LEARN! Research Institute, Vrije Universiteit Amsterdam, Amsterdam, Netherlands

**Keywords:** growth mindset, school burnout symptoms, physiological resilience, self-regulating strategies, vagal activity, heart rate variability

## Abstract

Experiencing school burnout symptoms can have negative consequences for learning. A growth mindset, the belief that human qualities such as intelligence are malleable, has previously been correlated with fewer school burnout symptoms in late adolescents. This might be because adolescents with a stronger growth mindset show more adaptive self-regulation strategies and thereby increasing resilience against academic setbacks. Here we confirmed in a sample of 426 Dutch young adolescents (11–14 years old; 48% female) that this relationship between growth mindset and school burnout symptoms holds after controlling for other potential predictors of school burnout symptoms such as academic achievement, school track, gender, and socio-economic status. Our second aim was to increase our understanding of the mechanism underlying the relation between mindset and school burnout, by measuring physiological resilience (vagal activity, a measure of parasympathetic activity, also known as heart rate variability or HRV) in a subsample (*n* = 50). We did not find any relation between vagal activity and growth mindset or school burnout symptoms, nor could we establish a mediating effect of vagal activity in their relation. In conclusion, we found evidence for a potential protective effect of a growth mindset on school burnout symptoms in young adolescents, but not for physiological resilience (vagal activity) as an underlying mechanism. The protective effect of growth mindset as confirmed in our younger sample can be leveraged in interventions to prevent increasing school burnout symptoms.

## 1. Introduction

Many studies in educational research focus on academic achievement rather than on psychological outcomes (Konu et al., 2002), even though these may be equally important aspects of academic functioning. A relevant negative psychological outcome is school burnout (Salmela-Aro et al., 2009). It can be considered as a continuous variable, ranging from school-related stress to major burnout (Salmela-Aro et al., 2008b). School burnout symptoms can be divided into three main dimensions: exhaustion at school because of school demands, school-related cynicism, and inadequacy at school (Salmela-Aro et al., 2009). Experiencing school burnout symptoms has negative effects on other school-related outcomes such as a decline in academic achievement, difficulties with concentration, lower problem-solving skills (May et al., 2015), and school drop-out (Bask and Salmela-Aro, 2013; Fiorilli et al., 2017). Because of these negative consequences, it is important to investigate factors that may protect against developing school burnout.

A factor that may protect against experiencing school burnout is resilience to academic setbacks and stressors (Yaghoobi and Baktiari, 1970; Fiorilli et al., 2020; Romano et al., 2021). From a physiological perspective, resilience is the adaptive maintenance of normal physiology, development, and behavior in the face of pronounced stress and adversity by means of allostasis (Pfau and Russo, 2015; Pereira et al., 2017). One factor that may influence adolescents’ resilience is their mindset. According to Dweck (1999), mindset refers to implicit beliefs concerning the malleability of one’s own qualities, such as intelligence, that can be described on a continuum. This continuum ranges from growth mindset (i.e., the belief that abilities or intelligence can be improved by your own actions) to fixed mindset (the belief that ability or intelligence is largely fixed and cannot change by your own actions). Mindset theory brings together goals, beliefs and behaviors in a system that gives meaning and interpretations to events, such as failure and setbacks during learning (Dweck and Yeager, 2019). People with a stronger growth mindset show more adaptive self-regulation strategies (Burnette et al., 2013), including more adaptive responses to setbacks (Dweck and Leggett, 1988; Aditomo, 2015). They are more likely to attribute failures to a lack of effort, which is under their own influence, while individuals with a stronger fixed mindset attribute failures to a lack of ability.

Interpretations of academic setbacks from a growth mindset meaning system show interesting similarities with resilience. When a person shows resilience, it does not mean that they do not perceive a situation as stressful, but that they are able to cope with it successfully (Feder et al., 2009; Walker et al., 2017). A recent meta-analysis concerning different types of mindsets (e.g., about intelligence and emotion) and psychological distress showed that individuals with stronger growth mindsets indeed view affective states as temporary rather than permanent. They embrace such feelings and cope with them effectively rather than avoiding them (Burnette et al., 2020). This meta-analysis also showed that individuals with a stronger growth (versus a fixed) mindset report lower psychological distress. In other words, these results suggest greater resilience in students with a stronger growth mindset.

Because of their greater resilience, growth mindset students may also be less vulnerable to develop school burnout symptoms. One study that directly investigated this relation between mindset and school burnout symptoms indeed showed that students with a stronger growth mindset reported lower school burnout symptoms (Kim, 2020). However, this was studied in 17-year-olds, while a study conducted among Finnish children suggests that school burnout symptoms already develop in early adolescence (Salmela-Aro et al., 2016). In the Netherlands there is an important transition from primary to secondary school in this early adolescence phase. This transition could be experienced as stressful and challenging, and mindset could play an important protective role against developing negative school-related outcomes during and after such challenging transitions (Blackwell et al., 2007). Therefore, it is important to understand the relation between mindset and school burnout symptoms in a younger sample that just made the challenging transition to secondary education.

Also, the role of possible relevant confounders remains unclear. The study of Salmela-Aro et al. (2008a), for example, showed that adolescents in an academic school track experience more exhaustion at school than adolescents in a vocational track. Furthermore, a recent study showed that lower academic achievement was associated with more school burnout symptoms (Herrmann et al., 2019) and that girls tend to report more school-related exhaustion than boys. Lastly, Luo et al. (2016) showed that socio-economic status (SES) had a negative direct effect the school burn-out symptoms emotional exhaustion and cynicism. Based on these previous studies it is likely that school burnout symptoms might also be influenced by factors such as, school track, academic achievement, gender, and SES. Further validation of the relationship between mindset and school burnout symptoms is necessary.

Another caveat in previous studies is that resilience is measured with subjective reports (Ahern et al., 2006). However, resilience is multifactorial. It not only includes many psychological constructs but also various physiological responses (Walker et al., 2017). A questionnaire may therefore not be the best way to capture resilience. A more objective measure of resilience could be vagal activity, or heart rate variability (HRV) (Laborde et al., 2017). Vagal activity reflects the activity of the vagus nerve, which delivers parasympathetic signaling to the heart using the neurotransmitter acetylcholine. Vagal activity is highly variable in response to stress (Castaldo et al., 2015; Kim et al., 2018) and is specifically important for facilitating the heart’s return to homeostasis after stress is averted (Porges, 1992, 1995, 2009; Porges et al., 1994; Janig and Habler, 2000). Moreover, according to the neurovisceral integration model by Thayer and Lane (2009), vagal activity can serve as an indicator of heart-brain interactions due to the bi-directional nature of the vagal fibers. Specifically, they believe that vagal activity can be used as a marker of prefrontal cortex inhibition. This makes it a very suitable marker for top-down processes related to emotion regulation (Li and Sinha, 2008). Such process might be related to resilient responses to academic failure and setbacks that are central in mindset theory. Their research has been supported by findings from various studies using two indices of vagal activity: vagal tone and vagal withdrawal. *Vagal tone* is a measure of vagus nerve activity at rest. Having a higher vagal tone is associated with better behavioral inhibition (Fabes and Eisenberg, 1997), more use of adaptive coping strategies (Geisler et al., 2010; Vögele et al., 2010; Geisler et al., 2013), less emotion regulation problems (Vasilev et al., 2009), higher trait resilience (Souza et al., 2013) and lower self-reported emotional arousal to conditions of moderate to high intensity stressors in daily life (Fabes and Eisenberg, 1997) in adolescents. *Vagal withdrawal* is the extent to which the vagus nerve decreases its activity when encountering a stressor. Higher vagal withdrawal in response to a stressor has been associated with less emotion regulation problems (Vasilev et al., 2009), less use of negative coping strategies (Fiol-Veny et al., 2019), higher perseverance (Silvia et al., 2013), and less experienced negative affect on a day-to-day basis in boys (Diamond et al., 2012) in adolescents.

Due to its relations with various emotion regulation constructs, self-regulated behaviors, and trait resilience, vagal activity is a good candidate to study the link between mindset and an objective physiological marker of resilience in the context of school-related stress and setbacks. Concretely, it is expected that students with a growth mindset are more resilient to school stress which will be reflected by a higher vagal tone and vagal withdrawal. Through this physiological resilience, these students experience less school burnout symptoms when confronted with academic stressors than students with fixed mindset. This idea of vagal activity as a *mediator* between mindset and school burnout would predict that the measures of vagal activity themselves are correlated to school burnout symptoms. Indeed, lower vagal tone and vagal withdrawal are associated with more emotion regulation problems, which have been associated with higher school burnout symptoms (Chacón-Cuberos et al., 2019; Chen, 2021; Vinter et al., 2021). To our knowledge only one study tried to link autonomic nervous system (ANS) activity to school burnout. May et al. (2018) could not link vagal activity to school burnout, but their data was collected during a 24-h unsupervised period, during which cardiac activity is influenced by factors such as stressors and movement. Especially the latter has a large effect on vagal tone (Fu and Levine, 2013) and was not corrected for in their analyses. No studies thus far have investigated the relationship between vagal activity and school burnout in a controlled, movement-free environment and with regard to academic stress reactivity.

**Figure 1:**
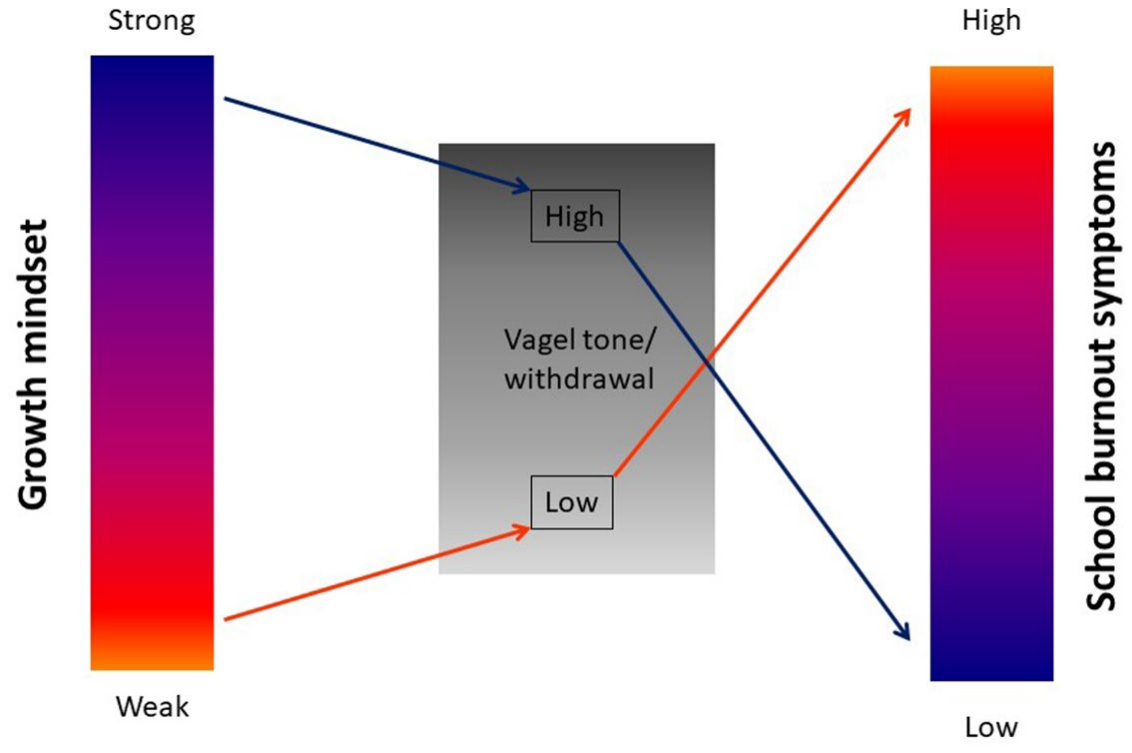
Visual representation of the hypothesized relations between mindset, vagal activity and school burnout symptoms.

To better understand the relation between mindset and school burnout symptoms and the underlying physiological mechanisms, the first aim of this study was to replicate and extend the findings of Kim (2020), where we hypothesize growth mindset to be negatively related to school burnout symptoms also in young adolescents. We controlled for school track, grade point average, self-identified gender, and SES to investigate if the expected relationship holds while controlling for these other factors. The second aim was to increase our mechanistic understanding of how mindset and school burnout symptoms are related by investigating the mediating effect of vagal activity. We hypothesized that endorsing a growth mindset would be positively associated with vagal activity, more specifically, higher baseline vagal tone and higher vagal withdrawal. We predicted a negative relationship between vagal activity and school burnout symptoms; thus, higher baseline vagal tone and vagal withdrawal should be linked to lower school burnout symptoms. Additionally, we tested whether vagal activity was a mediator in the relationship between mindset and school burnout (see Figure 1).

## 2. Materials and methods

### 2.1. Design

The analysis plan of study was pre-registered at the Open Science Framework.^1^ This cross-sectional study is part of a longitudinal study of which we only use the first measurement. All participants completed multiple questionnaires and were invited to join the laboratory study. During the laboratory study the participants came to the laboratory where we measured their vagal activity during rest and during a stressful mathematics task [the Math Effort Task (MET), see 2.4.4]. All procedures were approved by the local ethical committee of the faculty of Behavioral and Movement Sciences of de Vrije Universiteit Amsterdam and in line with the Declaration of Helsinki.

### 2.2. Participants

#### 2.2.1. Full sample

A total of 439 seventh-grade adolescents volunteered for the study. However, due to incomplete questionnaires the final sample consisted of 426 adolescents (48% self-identified as female). They were recruited from 20 classes of the first year of two secondary schools in the Netherlands. Age ranged between 11 and 14 years old (*M* = 12.72, *SD* = 0.48). See Table 1 for distribution among school tracks.

**Table 1:** Distribution adolescents among school tracks.

	Full sample		Subsample	
School track	*n*	%	*n*	%
Pre-vocational	40	9	2	4
General secondary education	81	19	4	8
Combination GSE and pre-university	119	28	12	24
Pre-university	102	24	5	10
Pre-university extra challenging	84	20	27	54

GSE, general secondary education.

#### 2.2.2. Subsample

All students included in the full sample were invited to participate in our additional lab study. Fifty-two participants were willing to participate, but due to incomplete questionnaire data the final sample consisted of 50 participants (50% female, age mean = 12.59, *SD* = 0.55). See Table 1 for distribution among school tracks.

#### 2.2.3. Exclusion criteria

There were no exclusion criteria for this study. All first-year students of the participating classes could participate if they wanted to and received informed consent from their parents.

### 2.3. Procedure

Several schools throughout the Netherlands were asked to participate in a larger longitudinal study. If the school was willing to participate, the participants were recruited via information letters that were sent to their parents. This information letter contained general information about the larger longitudinal intervention study (see Janssen and van Atteveldt, 2022), and about the current lab study that took place before the intervention and was a standalone, optional component of the study. For this lab study we asked whether participants and their parent(s)/caregiver(s) were willing to visit us in the lab to perform two computerized tasks when measuring their (electro)physiology with electrocardiography (ECG), impedance cardiography (ICG) and electroencephalography (EEG), of which only ECG and ICG were used in the current study.

When both the participants and their primary caregiver had provided active informed consent for the lab session, a link to the digital questionnaire with multiple motivational constructs and demographics was sent to the primary caregiver/parents of the participating adolescents to make sure their child filled out the questionnaire independently and in a quiet space. In this study we only analyzed the mindset and school burnout symptoms constructs. On average answering the questionnaire took 45 min. For the laboratory session, participants visited the university. They were tested individually in a laboratory room. After the electrodes were placed on the chest and back, they were seated in front of a computer screen where the session started. The session consisted of the following components: baseline recordings for vagal activity with eyes open staring at a fixation cross (5 min), the math effort task including academic stressor (MET, 20 min) and the stop-signal task (SST, 25 min; not used in this study).

### 2.4. Measures

#### 2.4.1. Mindset

Mindset was measured using a Dutch translated version of the revised self-theory scale designed by De Castella and Byrne (2015). This questionnaire consists of eight items, four items concerning a fixed mindset (example: “*To be honest, I do not think I can really change how intelligent I am*”) and four items concerning a growth mindset (example: “*I believe I can always substantially improve on my intelligence*”). Each item was scored on a Likert scale from 1 (*totally disagree*) to 6 (*totally agree*). The fixed mindset items were reversed and summed up with the growth mindset items to create one growth mindset variable. Higher sum scores indicated strong growth mindset endorsement. Scores ranged from 14 to 48 in the full sample, and from 18 to 46 in the subsample. Research indicates that the scale shows a good internal consistency, α = 0.90 (De Castella and Byrne, 2015). In this current study the internal consistency was good (α = 0.81 in the full sample and α = 0.85 in the subsample).

#### 2.4.2. School burnout symptoms

To measure participants’ school burnout symptoms, a Dutch translated version of the nine-item School Burnout Inventory (SBI; Salmela-Aro et al., 2009) was used. The scale consists of four ‘exhaustion at school’ items, three ‘cynicism towards the meaning of school’ items, and two ‘sense of inadequacy at school’-items. Examples of items for each subscale are “*I feel overwhelmed by my schoolwork*” for exhaustion at school, “*I feel that I’m losing interest in my schoolwork*” for cynicism towards the meaning of school, and “*I often have feelings of inadequacy in my schoolwork*” for sense of inadequacy at school. The extent in which participants agreed with the statements were indicated on a six-point Likert scale (1 = *Totally disagree* to 6 = *Totally agree*). A sum score was computed, in which a higher score indicated that participants experienced more school burnout symptoms. In the full sample scores ranged from 9 to 54 and in the subsample, scores ranged from 12 to 47. The internal consistency found in previous research was sufficient (α = 0.67–0.80; Salmela-Aro et al., 2009). In both current samples the internal consistency was good (*α* = 0.83 in the full sample and *α* = 0.85 in the subsample).

#### 2.4.3. Control variables full sample

##### 2.4.3.1. Gender

Self-identified gender was asked at the beginning of the questionnaire. We used this as covariate, because girls tend to report more exhaustion than boys (Herrmann et al., 2019).

##### 2.4.3.2. SES

An estimation of participants’ SES was made using the first four digits of the postal code of the participants’ home addresses. To this end, we used information available from a register at *Rijksinstituut voor Volksgezondheid en Milieu* (RIVM) provided data on SES scores (called status scores) for every four-digit postal code district in the Netherlands that has a minimum of one hundred households. The status scores in the RIVM register were realized through a principal component analysis (PCA) on districts’ average income, and percentages of low-income households, low educational attainment, and unemployment RIVM. A given status score represented the average SES of a four-digit postal code district, relative to other postal code districts. Estimating SES based on where students lived permitted us to include a reliable measure of SES without having to ask participants for more ethically sensitive personal information, such as parental income, educational level, or employment status. We included SES as a covariate because SES might be related to school burnout symptoms (Luo et al., 2016).

##### 2.4.3.3. School track

Dutch secondary education is tracked. There are seven tracks in total. While tracking can start from grade 7 on (age 12–13), many secondary schools have classes that are combinations of tracks in the lower grades. Participating adolescents were from one of the three upper tracks, a combination class or an extra challenge class of the upper track. For brevity, we refer to these five as tracks. They are summarized in Table 1. We included school track as covariate because a previous study showed that students on an academic track experienced more exhaustion than students in a vocational track (Salmela-Aro et al., 2008a,b).

##### 2.4.3.4. Grade point average

Adolescents’ academic achievement was measured by their Grade Point Average (GPA) at their school, including Dutch, English, Math, Geography, and History. The GPA scale in the Netherlands ranges from 1 (lowest) to 10 (highest). Adolescents’ grades were retrieved in the period around the completion of the questionnaire and reflected a grade from the beginning of the school year until the moment of the study. Note that in the Dutch school system, students’ achievement is graded relative to their school track. This means that students at the higher general secondary education need to meet higher standards to receive an 8 compared to pre-vocational education students, but they need to meet lower standards compared to students at the pre-university school track. We included GPA as covariate because lower academic achievement might be related to more school burnout symptoms (Herrmann et al., 2019).

#### 2.4.4. Math effort task

For the academic stressor (to enable measuring vagal withdrawal) we used an adapted version of the MET developed by Engle-Friedman et al. (2003), programmed in OpenSesame (Mathôt et al., 2012). In this arithmetic task students were presented with addition trials of three numbers, which were presented for 1,000 milliseconds each in a sequence (with 750 ms between the numbers), and which they were instructed to sum up (see Table 2). After the last number, participants had 5 s to type in their answer. The task started with two practice problems, followed by a first block with fixed addition trials with increasing difficulty (levels 1–5) to expose the participants to an academic stressor. In this block, three problems of each difficulty level were presented in a fixed order and with fixed number sequences to ensure everyone received the same difficulty. No performance feedback was given, again to avoid differences in stress exposure. After this first block, several blocks with self-adjusted difficulty were started (which are not part of this study). During the academic stressor block, vagal activity was continuously measured to assess vagal withdrawal (see section 2.4.5). The total duration of the academic stressor block was 3 min.

**Table 2:** Explanation of difficulty levels in the arithmetic task.

Difficulty level	Numbers
Level 1	Numbers between 1 and 3
Level 2	Numbers between 3 and 9
Level 3	Numbers between 7 and 15
Level 4	Numbers between 7 and 25
Level 5	Numbers between 7 and 35

#### 2.4.5. Vagal activity

Vagal activity was measured during the laboratory session with the 7-wire Vrije Universiteit Ambulatory Monitoring System (VU-AMS), version VU-AMS5fs, and analyzed using the corresponding VU-DAMS program.^2^ Seven adhesive 55 mm Kendall H98SG hydrogel ECG electrodes (Medtronic, Heerlen, Netherlands) were placed on the subject’s torso to record the ECG and ICG signals (as described in the VU-AMS manual, available at).^3^ ECG was sampled at 1 kHz and ICG at 250 Hz. Vagal activity was measured using Respiratory Sinus Arrhythmia (RSA, a common measure of Heart Rate Variability/HRV) applying the peak-to-valley method (De Geus et al., 1995) embedded in the VU-DAMS analyses program. This method combines the Inter-beat-interval (IBI) derived from the ECG with the respiration rate derived from the ICG, by subtracting the shortest IBI during inspiration from the longest IBI during expiration. The VU-DAMS software automatically detects the inspiration-expiration phase using an automatic scoring algorithm. Furthermore, the algorithm automatically detects periods containing artifacts such as: clipping (signal being out of range), irregular respirations (due to movement artifact), flat signal (to shallow breathing patterns), and missing data (electrode lost contact). When RSA has a negative value, or either the shortest inspiratory or longest expiratory IBI is missing, the RSA value for these breaths are considered zero. In addition to the software algorithms the signals were manually checked and adjusted if necessary. When more than 50% of a condition is erroneous this data was excluded from analysis. Regarding vagal activity we are interested in both vagal tone and vagal withdrawal. *Vagal tone* was defined as RSA during baseline recordings for vagal activity. *Vagal withdrawal* was defined as the RSA reactivity to the academic stressor by subtracting the baseline RSA from the RSA during the academic stressor.

When measuring vagal activity respiration rate was also measured. Respiration rate is a continuous measure derived from the impedance cardiogram. The VU-DAMS software automatically detects the inspiration-expiration phase using an automatic scoring algorithm. A single breath is defined as a full cycle containing both the inspiration and expiration. It is measured in breaths per minute by adding the total number of complete cycles during a 60 s fragment. We use the respiration rate during the baseline resting recordings and the reactivity measure of respiration rate during the academic stressor (respiration during academic stressor – respiration rate during baseline recordings). Because the relevance of adding respiration rate as a covariate for vagal activity is under discussion (Laborde et al., 2017), we present the findings of the study with and without respiration rate as covariate.

### 2.5. Analyses

#### 2.5.1. Descriptive statistics

We first calculated descriptive statistics and performed independent sample *t*-tests, to test for differences between the participants in the full sample and subsample.

#### 2.5.2. Full sample analysis

The hypothesis that growth mindset was negatively related to school burnout symptoms was tested using a hierarchical multiple regression analysis in the full sample, with the sum score of school burnout symptoms as the dependent variable. The primary predictor was the sum score of growth mindset. Therefore, growth mindset was entered in the first step of the regression analysis. Four variables were identified as relevant covariates (GPA, school track, gender, and SES). The covariates GPA and school track were considered as endogenous variables (variables that may have already been influenced by our motivational constructs) and were entered in the second step of the regression analysis. Gender and SES were considered as exogenous variables (variables that could not be influenced by our motivational constructs) and were entered in the third step of the regression analysis.

The model met the assumptions for the hierarchical multiple regression (normality and homoscedasticity). The intraclass correlation coefficient (ICC) design effect [1 + (*n* − 1)*ICC] was under 2 (Muthen and Satorra, 1995), which means there was no meaningful clustering at the class level and the hierarchical multiple regression analysis could be continued. We also performed sensitivity analyses to compute the minimal effect sizes that were detectable with the sample sizes in this study. With 426 participants, an alpha level of 0.05, a power of 0.80, and 5 predictors the minimal detectable effect size was small (*f*^2^ = 0.03).

#### 2.5.3. Subsample analyses (vagal activity)

We first performed a paired sample t-test, to investigate whether the stressor induced vagal withdrawal (academic stressor vs. baseline).

To answer whether growth mindset was related to vagal activity, we performed a multiple regression analysis in the subsample for which we obtained the physiological data. For the first part of the hypothesis, in which we predicted that mindset was related to baseline levels of vagal tone, baseline RSA (vagal tone) was entered as a dependent variable and growth mindset as an independent variable. For the second part of the hypothesis, in which we predicted that growth mindset was related to vagal withdrawal, RSA reactivity (vagal withdrawal; RSA stressor vs. baseline) was entered as the dependent variable. Due to differences in autonomic nervous system (ANS) stress reactivity between males and females (Huang et al., 2013), gender was also included as a covariate. Also, respiration rate was added as covariate because changes in respiration rate influences RSA, independent of changes in parasympathetic nervous system (PNS) activity (Grossman and Taylor, 2007). The same approach as for mindset versus vagal activity was used to answer the hypotheses regarding vagal activity and school burnout symptoms, only in these analyses school burnout symptoms was the dependent variable and baseline RSA (vagal tone) and RSA reactivity (vagal withdrawal) during the MET the independent variables. For the hypotheses regarding mindset and vagal activity and school burnout symptoms and vagal activity, we also performed a sensitivity analysis. For this subsample of 50 participants, the minimal detectable effect size was medium (*f*^2^ = 0.24), with an alpha level of 0.05, a power of 0.80 and three predictors.

To test whether vagal activity mediated the relation between mindset and school burnout symptoms, we used the PROCESS (Hayes, 2017) SPSS macro to test the mediation models. Mindset was inserted as the independent variable, and school burnout symptoms as the outcome variable. For the first part of the hypothesis, baseline RSA (vagal tone) was inserted as the mediator variable, while for the second part of the hypothesis, MET RSA reactivity (vagal withdrawal) was inserted as the mediator variable. We executed these analyses with model 4 (default model for mediation analyses) and 5,000 bootstraps. We wanted to estimate the indirect effect of vagal activity on the relation between mindset and school burnout symptoms, instead of using the classic mediation model of Baron and Kenny (1986). Therefore, the relation between X and Y does not have to be significant to continue the analysis (Shrout and Bolger, 2002).

For these two mediation models we also performed a sensitivity analysis. For the mediation analyses we have 50 participants. With four predictors, an alpha level of 0.05, and a power of 0.80, the minimal detectable effect size was medium (*f*^2^ = 0.27).

We used a value of *p* <0.05 as an indicator of significance. Effect sizes for regression analyses were considered small (*f*^2^ = 0.02), medium (*f*^2^ = 0.15) or large (*f*^2^ = 0.35). Analyses were performed in IBM SPSS Statistics for Windows (version 26).

## 3. Results

### 3.1. Descriptive statistics

In Table 3 the descriptive statistics of the relevant variables are presented. We also performed independent sample t-tests to investigate whether the subsample is representative for the larger sample. Table 3 showed that students in the subsample did not differ on growth mindset and school burnout symptoms. However, SES and GPA were higher in the subsample.

**Table 3:** Descriptive statistics of relevant predictor and outcome variables and differences between them.

	Full sample (*N* = 426)		Subsample (*n* = 50)			
	M	SD	M	SD	*t*	value of *p*
Growth mindset	34.86	6.24	36.10	6.57	1.56	0.120
School burnout symptoms	27.02	7.79	27.10	8.09	0.07	0.941
Socio-economic-status	0.29	0.94	0.65	0.93	2.78	0.006
Grade point average	6.66	0.67	6.84	0.75	1.99	0.047

Degrees of freedom = 418. M, mean; SD, standard deviation.

### 3.2. Mindset and school burnout symptoms

To test the hypothesis that endorsing a growth mindset was related to school burnout symptoms, while controlling for other relevant factors related to school burnout symptoms (gender, school track, GPA, SES), a hierarchical regression analysis was performed. This analysis revealed a significant negative relationship between growth mindset and school burnout symptoms, which was present both when analyzed in isolation (Step 1), and when covariates were added (Steps 2–4); see Table 4. The results in Table 4 show that there was also a negative relation between GPA and school burnout symptoms. The variables gender, SES, and school track were not related to school burnout symptoms. Mindset explained a unique variance of 4% based on the squared part correlation when accounting for the other factors in the model.

**Table 4:** The relationship between mindset and school burnout symptoms.

Variables	*b*	SE	*β*	*t*	Sig.	*R2*	*F* Change	Sig. *F*
Step 1:						0.05	22.44	<0.001
Growth mindset	−0.28	0.06	−0.23	−4.74	<0.001			
Step 2:						0.08	5.39	0.005
Growth mindset	−0.28	0.06	−0.23	−4.65	<0.001			
GPA	−1.57	0.55	−0.14	−2.85	0.005			
School track	0.50	0.30	0.08	1.69	0.092			
Step 3:						0.08	1.75	0.174
Growth mindset	−0.26	0.06	−0.21	−4.36	<0.001			
GPA	−1.86	0.57	−0.16	−3.26	0.001			
School track	0.51	0.32	0.08	1.61	0.108			
Gender	−1.44	0.77	−0.09	−1.87	0.062			
SES	0.03	0.42	0.00	0.07	0.941			

Dependent variable is school burnout symptoms. SES, socio-economic-status; GPA, grade point average.

### 3.3. Vagal activity

The mean vagal tone (RSA during baseline) was 96.91 (SD = 42.05). A paired-samples t-test showed that the stressor induced a small vagal withdrawal, *t*(49) = 2.23, *p* = 0.030, *d* = 0.31, with a mean vagal withdrawal (RSA stressor – baseline) of −9.12 (*SD* = 28.89).

To test the hypothesis that growth mindset was related to vagal activity, we performed two regression analyses. We could not identify a relation between growth mindset and baseline vagal tone, *b* = −0.46, *SE* = 0.82, *t* = −0.56, *p* = 0.577 or vagal withdrawal, *b* = 0.73, *SE* = 0.55, *t* = 1.34, *p* = 0.188. We analyzed this hypothesis again with two Bayesian regression analyses to better interpret the null findings. These analyses revealed moderate evidence (*BF*_10_ = 0.29; see Table 5 for Bayes Factor cut-off points) in favor of the null hypothesis of no relation between growth mindset and vagal tone. Adding respiration rate and gender as covariates increased the model fit relative to a fitting model (*BF*_10_ = 57.88; very strong evidence). However, with these covariates included there was still moderate evidence against a relation between mindset and vagal tone (*BF*_10_ = 0.32). In addition, we found moderate evidence against a relation between growth mindset and vagal withdrawal (*BF*_10_ = 0.30). When respiration rate and gender were added as covariates, overall model fit became better (*BF*_10_ = 98.32; very strong evidence); evidence against a relation between growth mindset and vagal reactivity was now merely anecdotal (*BF*_10_ = 0.63).

**Table 5:** Bayes factor cut-off points for interpretation.

Bayes factor (BF_10_)	Interpretation
>100	Extreme evidence for H_1_ compared to H_0_
30–100	Very strong evidence for H_1_ compared to H_0_
10–30	Strong evidence for H_1_ compared to H_0_
3–10	Moderate evidence for H_1_ compared to H_0_
1–3	Anecdotal evidence for H_1_ compared to H_0_
1	No evidence
1/3–1	Anecdotal evidence for H_0_ compared to H_1_
1/10–1/3	Moderate evidence for H_0_ compared to H_1_
1/30–1/10	Strong evidence for H_0_ compared to H_1_
1/100–1/30	Very strong evidence for H_0_ compared to H_1_
<1/100	Extreme evidence for H_0_ compared to H_1_

Bayes factor cut-off points are based on the interpreting guidelines of Lee and Wagenmakers (2014).

Next, we tested the hypothesis that vagal activity was related to school burnout symptoms. We could not identify a relationship between baseline vagal tone and school burnout symptoms, (*b* = 0.02, *SE* = 0.03, *t* = 0.52, *p* = 0.609 or between vagal withdrawal and school burnout symptoms, *b* = −0.01, *SE* = 0.05, *t* = −0.26, *p* = 0.795). Also, here Bayesian regression analyses were performed to interpret the null results. We found moderate evidence in favor of the null hypothesis indicating no relation between vagal activity and school burnout symptoms (vagal tone: *BF*_10_ = 0.30; vagal withdrawal: *BF*_10_ = 0.29). Adding the covariates respiration rate and gender did not improve the model fit in both models. However, the evidence against a relation between vagal tone and school burnout symptoms became anecdotal (*BF*_10_ = 0.43). Also, the evidence against a relation between vagal withdrawal and school burnout symptoms became anecdotal (*BF*_10_ = 0.41).

We noted that vagal withdrawal showed a large variability (range = −102.97 to 42.20). Of the 50 participants 17 showed a higher vagal activity during the academic stressor compared to the baseline, i.e., no withdrawal. Exploratory independent sample t-tests comparing this group to the adolescents that did show the expected vagal withdrawal revealed no significant differences in growth mindset, school burnout, age, GPA, SES, baseline vagal tone, or vagal activity levels during the academic stressor. To ascertain that the observations, specifically for those adolescents who showed an increase in RSA in response to the stressor, were not due to respiration artifacts, we verified our analyses by repeating all frequentist analyses but then with heart rate (HR). HR is more robust to respiration rate and is therefore less sensitive to artifacts. These analyses with HR were not different from the analyses with RSA (see Supplementary Material S1–S4).

Even though there was no significant relation between vagal activity and school burnout, we still performed both the pre-registered and hypothesized mediation analyses. The mediation analyses showed that there was no direct effect of mindset on school burnout symptoms (model 1 with baseline vagal tone: *b* = −0.23, *SE* = 0.18, *t* = −1.24, *p* = 0.223; model 2 with vagal reactivity: *b* = −0.24, *SE* = 0.18, *t* = −1.31, *p* = 0.19). Vagal activity did not mediate the relation between mindset and school burnout symptoms in both models (baseline vagal tone: *b* = 0.01, *SE* = 0.04, *Bootstrap CI* [−0.09–0.08]; vagal reactivity: *b* = −0.00, *SE* = 0.05, *Bootstrap CI* [−0.09–0.13]). In both analyses we controlled for gender and respiration rate.

## 4. Discussion

The first aim of this study was to extend the previous findings of Kim (2020), namely the relation between growth mindset and school burnout symptoms, in a sample of younger adolescents who have just made the challenging transition to secondary school. We aimed to investigate whether a protective effect of growth mindset on school burnout symptoms can be found at a younger age and holds after controlling for other relevant factors for developing school burnout symptoms, such as gender, SES, GPA and school track. The second aim of this study was to increase our mechanistic understanding of how mindset and school burnout symptoms are related by investigating the mediating effect of vagal activity as physiological marker of resilience in a subsample.

### 4.1. Mindset and school-burnout symptoms

In line with Kim (2020) findings in late adolescence we found that young adolescents with a stronger growth mindset reported lower school burnout symptoms. Although the effect was small, this effect remained even when we controlled for other possible factors that have been related to school burnout symptoms in previous research, such as gender, GPA, SES, and school track (Salmela-Aro et al., 2008a,b; Luo et al., 2016; Herrmann et al., 2019). Next to mindset, GPA was also negatively related to school burnout symptoms: adolescents with lower average grades reported higher school burnout symptoms. This is in line with previous literature that found that lower school achievement was associated with more school burnout symptoms (Herrmann et al., 2019). Gender, SES, and school track were, in contrast to the literature, not related to school burnout symptoms. These findings imply that already early in secondary school adolescents benefit from having a stronger growth mindset, as it is associated with lower negative academic outcomes. Since this study is cross-sectional, future studies should investigate if the relation between growth mindset and school burnout symptoms is causal. Longitudinal studies could provide stronger claims about the possible protective effects of growth mindset in developing school burnout symptoms. This could be helpful in targeting early interventions to make adolescents more resilient to academic setbacks and stressors to prevent the increase of school burnout symptoms. A recent study showed that adolescents who received a growth mindset intervention showed higher math grades a year after the intervention (Janssen and van Atteveldt, 2022). This effect was relatively large (*d* = 0.36) compared to the literature (see Sisk et al., 2018). Nevertheless, Janssen and van Atteveldt (2022) could not establish a diminishing effect of the growth mindset intervention on school burnout symptoms, which might take longer to manifest. In a recent two-year follow-up study of the same intervention (Janssen and van Atteveldt, 2023), a relation was found between induced growth mindset and resilience against school burnout symptoms during COVID-19 that was fully mediated by the use of more adaptive coping strategies. This might implicate that coping is an important mechanism trough which growth mindset could influence resilient behavior. Another interesting perspective is related to cognitive load theory. A study by Xu et al. (2021) suggested that students with a stronger growth mindset experience lower cognitive load. It could be speculated that experiencing lower levels of cognitive load, might in turn lead to lower exhaustion levels and feeling of inadequacy that are related to school burnout symptoms. If future research will confirm this hypothesis, it might have implications for feedback and instruction that could be focused on autonomy-supportive practices which are related to the development of growth mindset (Yu et al., 2022).

### 4.2. Vagal activity

To assess the second aim, we investigated in a subsample whether the relation between mindset and school burnout symptoms can be explained by vagal activity. In our subsample we did not find any relations between vagal activity, growth mindset and school burnout symptoms. Also, in contrast to our predictions there was no indirect effect of vagal activity in the relation between mindset and school burnout symptoms. The Bayesian analyses revealed that there was moderate to anecdotal evidence in favor of the null hypothesis (no effect of vagal activity on growth mindset and school burnout symptoms). This means that our results are inconclusive, as there is neither strong evidence for no effects, nor strong evidence in favor of any effects of vagal activity.

This lack of effect could be a result from the low level of stress induced by the math task, and that an effect will only be present under more stressful conditions. Specifically in relation to mindset and school burnout, it might be more relevant to study the relationship in a more ecological valid context such as an actual school exam. We therefore believe that vagal activity is still of interest as a potential mechanism on how beliefs such as mindset may exert influence on school burnout symptoms. Specifically, since it provides insights complementary to self-report. For example, researchers already questioned the accuracy of children’s (4–13 years old) reflections of their own motivated behavior (Nicholls, 1978; Pintrich and Schunk, 2002). An objective measure of resilience, such as vagal activity, could therefore present a more accurate measure of mechanisms underlying motivated behavior, including mindset, especially in this age group. Below we discuss several limitations and suggestions for how to address these to find more conclusive evidence in future research.

### 4.3. Strengths and limitations

A strong point of this study is that we have combined different methods to assess the relation between mindset and school burnout symptoms. Previous studies already suggest that such integration of methods leads to a more thorough analysis of the concepts that are measured in educational psychology and should be conducted more often (Fulmer and Frijters, 2009; Vu et al., 2021).

We believe that our inconclusive findings regarding vagal activity are partly related to the following limitations. First, the distribution of growth mindset and school burnout symptoms was slightly negatively skewed. This resulted in a sample where most adolescents are on the growth mindset side of the continuum and showed relatively little school burnout symptoms. As a consequence, our findings cannot provide us with conclusions on the relationship between mindset and school burnout and their relationship to vagal activity within the complete continuum. It might be possible to establish a relation between vagal activity and mindset and school burnout symptoms when students have a stronger fixed mindset and more severe school burnout symptoms.

The second limitation was that our sensitivity analyses indicated that we only had enough power to detect medium to large effects. Although it is not uncommon in vagal activity research to find medium effects (Brindle et al., 2014), studies investigating mindset generally find small effects (Sisk et al., 2018). It is therefore possible that our subsample was underpowered.

Third, the subsample was influenced by self-selection. Only a small proportion of adolescents in the total sample agreed to participate in the additional laboratory session, resulting in a less representative subsample. The adolescents in the subsample came from a relatively higher socio-economic background compared to the average of the full sample and had on average a better academic performance. The range of values in growth mindset and school burnout symptoms might be somewhat restricted through this oversampling of high achieving students.

Fourth, as mentioned our stressor only induced a small decrease in vagal activity, which is lower compared to other known social or mental stressors (Brindle et al., 2014). A subgroup of adolescents even exhibited higher vagal activity and lower HR during the stressor compared to the baseline. The current task might therefore not have induced sufficient stress to differentiate on the basis of growth mindset endorsement or school burnout symptoms. This scenario is supported by the nature of our stressor, which may have been too predictable in the sense that the participants knew that the difficulty level would increase. Also, they did not receive performance feedback after each equation. This means they did not know whether they made a mistake or not. This could have resulted in less variation in vagal activity because the stressor was not strong enough and maybe especially for this subgroup which consists of high achievement adolescents with more beneficial backgrounds.

For future research to tackle this more effectively, it will be important to (1) have sufficient power to detect the smaller effects of vagal activity, (2) have a representative sample in terms of mindset, school burnout and academic achievement distributions, (3) use a stronger stressor to find more robust vagal withdrawal effects. To tackle the first two points a larger and more representative sample should be achieved by including more students from a vocational school track and lower social economic status (SES). This is especially important because research shows that specifically students with lower SES benefit from having a growth mindset (Yeager and Dweck, 2012). To tackle the third point, a stronger stress paradigm should be used, such as a social evaluative stressor (Yeager et al., 2022). Another option is to measure students on the day of a school exam in the naturalistic school environment to provide a more ecological valid stressor. Especially the latter is a promising approach to further investigate the mechanisms underlying a protective effect of growth mindset on real-life stress and becomes increasingly feasible with the development of wearable ECG devices.

## 5. Conclusion

In conclusion, this study aimed to gain more knowledge about the relation between mindset and school burnout symptoms. The study confirms previous findings in older adolescent in a sample of younger adolescents who just transitioned from primary to secondary education. In both phases of adolescence, a stronger growth mindset is related to lower school burnout symptoms. Moreover, this was found even when controlling for other potentially relevant factors for developing school burnout symptoms (GPA, school track, gender, and SES). We did not find evidence for physiological resilience (vagal activity) as an underlying mechanism in the relation between growth mindset and school burnout symptoms. However, as this one of the first attempts to unravel such a mechanism using more objective, physiological measures, the discussed limitations provide valuable recommendations for future research into these mechanisms.

## Data availability statement

Data and analysis code for this study are available by emailing the corresponding author.

## Ethics statement

The studies involving human participants were reviewed and approved by Vaste Commissie Wetenschap en Ethiek (VCWE), Faculty of Behavioral and Movement Sciences (FBM). Written informed consent to participate in this study was provided by the participants’ legal guardian/next of kind into primary caregiver.

## Author contributions

SN, TJ, and NA contributed to the conception and design. SN and LV wrote the first draft of the manuscript in a preliminary stage. SN has rewritten and added sections to the manuscript and performed data-analyses. DM wrote the paragraphs regarding resilience and vagal activity and performed data preprocessing activities and supported with data-analysis. TJ, MM, and NA have contributed by supervising all processes during the writing and data-analysis stages. All authors contributed to the article and approved the submitted version.

## Funding

This research was supported by European Research Council Starting grant 716736 (BRAINBELIEFS) to NA.

## Conflict of interest

The authors declare that the research was conducted in the absence of any commercial or financial relationships that could be construed as a potential conflict of interest.

## Publisher’s note

All claims expressed in this article are solely those of the authors and do not necessarily represent those of their affiliated organizations, or those of the publisher, the editors and the reviewers. Any product that may be evaluated in this article, or claim that may be made by its manufacturer, is not guaranteed or endorsed by the publisher.

## Footnotes

^1^https://osf.io/92s3r/

^2^https://vu-ams.nl/software-solutions/

^3^https://vu-ams.nl/support/
