# Supplementary material for: Growth mindset and school burnout symptoms in young adolescents: the role of vagal activity as potential mediator
Source: Front Psychol. 2023 Jul 13;14:1176477. doi: 10.3389/fpsyg.2023.1176477 (PMC10374320; doi:10.3389/fpsyg.2023.1176477)
Supplement: Supplementary file 1 [file Data_Sheet_1.docx]

Supplementary Material

# Supplementary tables

**Supplementary Table 1.**

*The effect of mindset on resting levels of Heart Rate while controlling for (self-identified) gender*

|  | *b* | *SE* | *β* | *t* | *p-value* |
| --- | --- | --- | --- | --- | --- |
| Gender | 2.66 | 2.33 | 0.16 | 1.14 | .261 |
| Growth mindset | 0.61 | 0.18 | 0.05 | 0.34 | .737 |

**Supplementary Table 2.**

*The effect of mindset on Heart Rate (HR) during the task while controlling for HR at rest and (self-identified) gender*

|  | *b* | *SE* | *β* | *t* | *p-value* |
| --- | --- | --- | --- | --- | --- |
| HR at rest | 0.96 | 0.10 | 0.83 | 9.67 | < .001 |
| Gender | -2.61 | 1.60 | -0.14 | -1.63 | .110 |
| Growth mindset | -0.21 | 0.12 | -0.15 | -1.74 | .089 |

**Supplementary Table 3.**

*The effect of Heart Rate at rest on school burnout symptoms, while controlling for (self-identified) gender*

|  | *b* | *SE* | *β* | *t* | *p-value* |
| --- | --- | --- | --- | --- | --- |
| Gender | 0.08 | 2.37 | 0.01 | 0.03 | .974 |
| HR Rate at rest | -0.01 | 0.15 | -0.01 | -0.10 | .925 |

**Supplementary Table 4.**

*The effect of Heart Rate (HR) during the task on school burnout symptoms, while controlling for HR at rest and (self-identified) gender*

|  | *b* | *SE* | *β* | *t* | *p-value* |
| --- | --- | --- | --- | --- | --- |
| HR at rest | 0.14 | 0.25 | 0.15 | 0.58 | .568 |
| Gender | -0.35 | 2.44 | -0.02 | -0.14 | .888 |
| HR during task | -0.17 | 0.21 | -0.19 | -0.78 | .438 |
